# Use of Artificial Intelligence Chatbots for Cancer Treatment Information

**DOI:** 10.1001/jamaoncol.2023.2954

**Published:** 2023-08-24

**Authors:** Shan Chen, Benjamin H. Kann, Michael B. Foote, Hugo J. W. L. Aerts, Guergana K. Savova, Raymond H. Mak, Danielle S. Bitterman

**Affiliations:** 1 Artificial Intelligence in Medicine Program, Mass General Brigham, Harvard Medical School, Boston, Massachusetts; 2 Department of Medicine, Memorial Sloan Kettering Cancer Center, New York, New York; 3 Computational Health Informatics Program, Boston Children’s Hospital, Boston, Massachusetts

## Abstract

This survey study examines the performance of a large language model chatbot in providing cancer treatment recommendations that are concordant with National Comprehensive Cancer Network guidelines.

Large language models (LLMs) underlying chatbots^1^ can mimic human language and quickly return detailed and coherent-seeming responses. These properties can obscure that chatbots might provide inaccurate information. Because patients often use the internet for self-education,^2^ some will undoubtedly use LLM chatbots to find cancer-related medical information, which could be associated with the generation and amplification of misinformation. We evaluated an LLM chatbot’s performance to provide breast, prostate, and lung cancer treatment recommendations concordant with National Comprehensive Cancer Network (NCCN)^3^ guidelines.

## Methods

We developed 4 zero-shot prompt templates to query treatment recommendations (eMethods and eFigure in Supplement 1). These templates do not provide the model with examples of correct responses. Templates were used to create 4 prompt variations for 26 diagnosis descriptions (cancer types with or without relevant extent of disease modifiers) for a total of 104 prompts. Prompts were input to the GPT-3.5-turbo-0301 model via the ChatGPT (OpenAI) interface. In accordance with the Common Rule, institutional review board approval was not needed since human participants were not involved.

We benchmarked the chatbot’s recommendations against 2021 NCCN guidelines because this chatbot’s knowledge cutoff was September 2021. Five scoring criteria were developed to assess guideline concordance (Table 1). The output did not have to recommend all possible regimens to be considered concordant; the recommended treatment approach needed only to be an NCCN option. Concordance of the chatbot output with NCCN guidelines was assessed by 3 of 4 board-certified oncologists, and majority rule was taken as the final score. In cases of complete disagreement, the oncologist who had not previously seen the output adjudicated. Data were analyzed between March 2 and March 14, 2023, using Excel, version 16.74 (Microsoft Corp).

**Table 1.** Oncologist Scoring of LLM Chatbot Treatment Recommendations^a^

Scoring criteria	Agreement across 4 unique prompts, No. (%) (n = 26)^b^	All prompts, No. (%) (n = 104)	Prompts by cancer type					Prompts by extent of disease		
			Breast cancer, No. (%) (n = 20)	Lung cancer, No. (%) (n = 20)^c^	Non–small cell lung cancer, No. (%) (n = 20)	Small cell lung cancer, No. (%) (n = 12)	Prostate cancer, No. (%) (n = 32)	Not specified, No. (%) (n = 20)	Localized, No. (%) (n = 64)	Advanced, No. (%) (n = 20)
**1. How many treatment recommendations were provided?**										
0	22 (84.6)	3 (2.9)	1 (5.0)	1 (5.0)	0	0	1 (3.1)	0	3 (4.7)	0
1		2 (1.9)	0	0	0	0	2 (6.3)	0	2 (3.1)	0
≥1		99 (95.2)	19 (95.0)	19 (95.0)	20 (100)	12 (100)	29 (90.6)	20 (100)	59 (92.2)	20 (100)
**2. How many of the recommended treatments were in accordance with 2021 NCCN guidelines?** ^c^										
0	9 (34.6)	0	0	0	0	0	0	0	0	0
Some but not all		35 (33.7)	2 (10.0)	6 (30.0)	8 (40.0)	7 (58.3)	12 (37.5)	2 (10.0)	26 (40.6)	7 (35.0)
All		66 (63.5)	17 (85.0)	13 (65.0)	12 (60.0)	5 (41.7)	19 (59.4)	18 (90.0)	35 (54.7)	13 (65.0)
NA		3 (2.9)	1 (5.0)	1 (5.0)	0	0	1 (3.1)	0	3 (4.7)	0
**3. If some but not all to question 2, how many recommendations were correct in their entirety per 2021 NCCN guidelines?**										
0	11 (42.3)	1 (0.9)	0	0	0	1 (8.3)	0	0	1 (1.6)	0
≥1		33 (31.7)	1 (5.0)	7 (35.0)	8 (40.0)	6 (50.0)	11 (34.4)	2 (10.0)	25 (39.1)	6 (30.0)
NA^d^		70 (67.3)	19 (95.0)	13 (65.0)	12 (60.0)	5 (41.7)	21 (65.6)	18 (90.0)	38 (59.4)	14 (70.0)
**4. How many recommended treatments were hallucinated?**										
0	16 (61.5)	91 (87.5)	19 (95.0)	18 (90.0)	19 (95.0)	9 (75.0)	26 (81.3)	19 (95.0)	55 (85.9)	17 (85.0)
≥1		13 (12.5)	1 (5.0)	2 (10.0)	1 (5.0)	3 (25.0)	6 (18.8)	1 (5.0)	9 (14.1)	3 (15.0)
**5. If >1 to question 4, how many of the hallucinated treatments are now a recommended treatment in the most current NCCN guidelines?**										
0	16 (61.5)	13 (12.5)	1 (5.0)	2 (10.0)	1 (5.0)	3 (25.0)	6 (18.8)	1 (5.0)	9 (14.1)	3 (15.0)
≥1		0	0	0	0	0	0	0	0	0
NA		91 (87.5)	19 (95.0)	18 (90.0)	19 (95.0)	9 (75.0)	26 (81.3)	19 (95.0)	55 (85.9)	17 (85.0)

Abbreviations: LLM, large language model; NA, not applicable; NCCN, National Comprehensive Cancer Network.

^a^ Data reported as No. (%) using majority rule of annotators’ scores.

^b^ Diagnosis descriptions for which the output of each of the 4 prompts for a given diagnosis description yielded the same score by the rating oncologists.

^c^ Lung cancer was queried separately from non–small cell lung cancer and small cell lung cancer.

^d^ Slight misalignment of categorical scores from questions 2 and 3 resulted from majority rules. For example, for question 3, NA = 70 instead of 69 (66 + 3) because of majority voting.

## Results

Outputs of 104 unique prompts were scored on 5 criteria for a total of 520 scores. All 3 annotators agreed for 322 of 520 (61.9%) scores. Disagreements tended to arise when the output was unclear (eg, not specifying which multiple treatments to combine). Table 1 shows agreement between the 4 prompts and the distribution of scores across cancer type and extent of disease. For 9 of 26 (34.6%) diagnosis descriptions, the 4 prompts yielded the same scores for each of the 5 scoring criteria. Table 2 shows scores across the prompts. The chatbot provided at least 1 recommendation for 102 of 104 (98%) prompts. All outputs with a recommendation included at least 1 NCCN-concordant treatment, but 35 of 102 (34.3%) of these outputs also recommended 1 or more nonconcordant treatments.

**Table 2.** Scoring of LLM Chatbot Treatment Recommendations According to Prompt Template^a^

Scoring criteria	Prompt template			
	What is a recommended treatment for [diagnosis description] according to NCCN? (n = 26)	What is a recommended treatment for [diagnosis description]? (n = 26)	How do you treat [diagnosis description]? (n = 26)	What is the treatment for [diagnosis description]? (n = 26)
**1. How many treatment recommendations were provided?**				
0	2 (7.7)	0	0	1 (3.8)
1	2 (7.7)	0	0	0
≥1	22 (84.6)	26 (100)	26 (100)	25 (96.2)
**2. How many of the recommended treatments were in accordance with 2021 NCCN guidelines?**				
0	0	0	0	0
Some but not all	5 (19.2)	11 (42.3)	11 (42.3)	8 (30.8)
All	19 (73.1)	15 (57.7)	15 (57.7)	17 (65.4)
NA	2 (7.7)	0	0	1 (3.8)
**3. If some but not all to question 2, how many recommendations were correct in their entirety per 2021 NCCN guidelines?**				
0	0	0	1 (3.8)	0
≥1	5 (19.2)	11 (42.3)	10 (38.5)	7 (26.9)
NA^b^	21 (80.8)	15 (57.7)	15 (57.7)	19 (73.1)
**4. How many recommended treatments were hallucinated?**				
0	25 (96.2)	23 (88.5)	23 (88.5)	20 (76.9)
≥1	1 (3.8)	3 (11.5)	3 (11.5)	6 (23.1)
**5. If **≥**1 to question 4, how many of the hallucinated treatments are now a recommended treatment in the most current NCCN guidelines?**				
0	1 (3.8)	3 (11.5)	3 (11.5)	6 (23.1)
≥1	0	0	0	0
NA	25 (96.2)	23 (88.5)	23 (88.5)	20 (76.9)

Abbreviations: LLM, large language model; NA, not applicable; NCCN, National Comprehensive Cancer Network.

^a^ Data reported as No. (%) using majority rule of annotators’ scores.

^b^ Slight misalignment of categorical scores from questions 2 and 3 resulted from majority rules.

Responses were hallucinated (ie, were not part of any recommended treatment) in 13 of 104 (12.5%) outputs. Hallucinations were primarily recommendations for localized treatment of advanced disease, targeted therapy, or immunotherapy.

## Discussion

One-third of treatments recommended by the chatbot were at least partially nonconcordant with NCCN guidelines; recommendations varied based on how the question was posed. Disagreement among annotators highlighted the challenges of interpreting descriptive LLM output. Disagreements most often arose from unclear output, but differing interpretations of guidelines among annotators may have played a role. Clinicians should advise patients that LLM chatbots are not a reliable source of treatment information.

Language learning models can pass the US Medical Licensing Examination,^4^ encode clinical knowledge,^5^ and provide diagnoses better than laypeople.^6^ However, the chatbot did not perform well at providing accurate cancer treatment recommendations. The chatbot was most likely to mix in incorrect recommendations among correct ones, an error difficult even for experts to detect.

A study limitation is that we evaluated 1 model at a snapshot in time. Nonetheless, the findings provide insight into areas of concern and future research needs. The chatbot did not purport to be a medical device, and need not be held to such standards. However, patients will likely use such technologies in their self-education, which may affect shared decision-making and the patient-clinician relationship.^2^ Developers should have some responsibility to distribute technologies that do not cause harm, and patients and clinicians need to be aware of these technologies’ limitations.

## Notes

Supplement 1.
eMethods
**eFigure.** Experimental Design

Supplement 2. Data Sharing Statement
