# Supplementary material for: Use of Artificial Intelligence Chatbots for Cancer Treatment Information
Source: JAMA Oncol. 2023 Aug 24;9(10):1459–62. doi: 10.1001/jamaoncol.2023.2954 (PMC10450584; doi:10.1001/jamaoncol.2023.2954)
Supplement: Supplement 1. — eMethods eFigure. Experimental Design [file jamaoncol-e232954-s001.pdf]

Supplemental Online Content

Chen S, Kann BH, Foote MB, et al. Utility of artificial intelligence chatbots for cancer treatment information. *JAMA Oncol.* Published online August 24, 2023. doi:10.1001/jamaoncol.2023.2954

eMethods

eFigure. Experimental Design

This supplemental material has been provided by the authors to give readers additional information about their work.

© 2023 Chen S et al. *JAMA Oncology.*

eMethods.

All of the prompts, outputs from ChatGPT, scores, and scoring guidelines are open-source and available to the public[1]. We have made our data easily accessible in a Google sheet. This means anyone can access and filter the responses based on their preferences and interests. Please note that the value set for the scores are available in the scoring guidelines.

For instance, if you are interested in understanding ChatGPT's treatment recommendations for different health conditions, you can simply filter the responses by the **"Prompt Template"** column to see the differences in the recommendations given.

Additionally, suppose you want to learn more about how ChatGPT responds to specific cancer types and stages. In that case, you can filter through the **"Cancer type"**, **"Extent of Disease"**, or **"Disease Description"** columns for more detailed and personalized information.

We encourage you to take advantage and read through the prompts with corresponding responses from ChatGPT.

[1] All resources are available at: https://github.com/AIM-Harvard/ChatGPT_NCCN

© 2023 Chen S et al. *JAMA Oncology.*

eFigure. Experimental Design

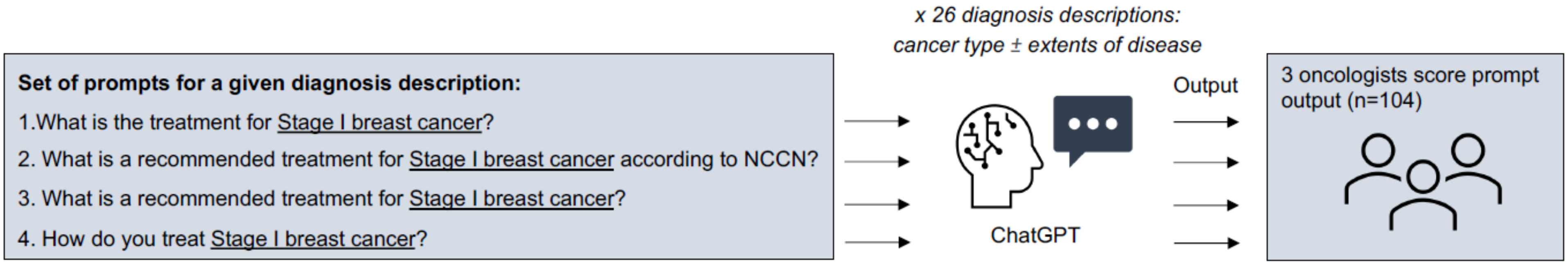

Underlined text indicates where each diagnosis description was input into the LLM chatbot prompt template. Diagnosis descriptions consisted of cancer type (breast cancer, lung cancer, non–small cell lung cancer, small cell lung cancer, and prostate cancer) with and without extents of disease relevant for each cancer type. A total of 26 disease descriptions were input into the prompt templates, for a total of 104 unique prompts. NCCN indicates National Comprehensive Cancer Network.

© 2023 Chen S et al. *JAMA Oncology.*
