# Supplementary material for: Use of Artificial Intelligence Chatbots for Cancer Treatment Information
Source: JAMA Oncol. 2023 Aug 24;9(10):1459–62. doi: 10.1001/jamaoncol.2023.2954 (PMC10450584; doi:10.1001/jamaoncol.2023.2954)
Supplement: Supplement 2. — Data Sharing Statement [file jamaoncol-e232954-s002.pdf]

Data Sharing Statement

Chen. Use of Artificial Intelligence Chatbots for Cancer Treatment Information. *JAMA Oncol*. Published August 24, 2023. doi:10.1001/jamaoncol.2023.2954

Data
Data available: Yes
Data types: Data (not involving human participants)
How to access data: All data, including prompts, ChatGPT output, and scoring guidelines are made available through the project github: https://github.com/AIM-Harvard/ChatGPT_NCCN
When available: With publication

Supporting Documents
Document types: Other (please specify)
Additional Information: All data used in this study and scoring guidelines are made available at the project github: https://github.com/AIM-Harvard/ChatGPT_NCCN
How to access documents: All data used in this study and the scoring guidelines are made available at the project github: https://github.com/AIM-Harvard/ChatGPT_NCCN
When available: beginning date: 03-15-2023

Additional Information
Who can access the data: Any researchers who would like to use the data.
Types of analyses: Any purpose.
Mechanisms of data availability: Without investigator support.
Any additional restrictions: No restrictions.
